# Effects of LAIR‐1 on hepatocellular carcinoma cell proliferation and invasion via PI3K‐AKT‐mTOR pathway regulation

**DOI:** 10.1002/iid3.982

**Published:** 2023-08-29

**Authors:** Ti Zhou, Luqing Liu, Haibin Lan, Donglin Fang

**Affiliations:** ^1^ Department of General Surgery The First People's Hospital of Lin ping District Hangzhou Zhejiang China; ^2^ Department of General Surgery The People's Hospital of Guannan County Lianyungang Jiangsu China

**Keywords:** HCC, LAIR‐1, pathway, PI3K‐AKT‐mTOR pathway

## Abstract

**Introduction:**

Hepatocellular carcinoma (HCC) is one of the common malignant tumors. Although surgical resection is the best treatment for HCC, many patients with HCC are found to have metastases at the time of initial diagnosis and lose the opportunity for radical treatment. Therefore, the study of the invasion and metastasis of HCC has always been the focus of HCC research. This study aimed to assess the influence of LAIR‐1 on HCC cell proliferation and invasion and the relevant mechanisms involved in this process.

**Methods:**

Immunocytochemical staining assay, quantitative real‐time polymerase chain reaction (qRT‐PCR) and western blotting (WB) were used to detect the expression of LAIR‐1mRNA and protein in healthy human hepatocyte LO2 and the HCC cell lines HepG2, Bel‐7402, MHCC97‐H, and Huh‐7. Then, we evaluated the cell viability, colony formation, and invasion of MHCC97‐H and Huh‐7 cells in each group by silencing or overexpressing LAIR‐1 expression in MHCC97‐H and Huh‐7 cells, respectively. WB was used to detect the expression levels of PI3K‐AKT‐mTOR pathway related proteins.

**Results:**

Our findings showed that LAIR‐1 can inhibit cell viability, colony formation and invasion in vitro. Meanwhile, LAIR‐1 significantly downregulated the expression of PI3K, p‐AKT and p‐mTOR, which were abolished by the PI3K inhibitor, LY294002.

**Conclusions:**

Our study revealed that LAIR‐1 inhibited cell proliferation and invasion, probably via suppressing the PI3K‐AKT‐mTOR pathway.

## Graphical abstract

This study first indicated that LAIR‐1 overexpression was detected in hepatocellular carcinoma (HCC) cells, and the downregulation of LAIR‐1 levels increased HCC cell viability, colony formation, and invasion, which might be associated with the PI3K‐AKT‐mTOR signaling pathway suppression.

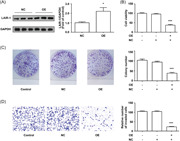

## 1 INTRODUCTION

Liver cancer is the third major cause of cancer‐relevant deaths and is the sixth most prevalent neoplasm.^1^ Approximately 72% of liver cancer cases happen in Asia, with over 50% in China.^2^ Hepatocellular carcinoma (HCC) is the most prevalent liver cancer and accounts for about 90% of all cases.^3^ In addition, HCC is the most aggressive and lethal liver cancer.^4^ HCC incidence rises rapidly, with over 600,000 deaths yearly.^5^

Currently, hepatectomy is the first choice for HCC treatment. Nonetheless, in the United States, the liver cancer 5‐year survival rate is only 18%.^6^ However, even after radical resection, the 5‐year survival rate of HCC patients rarely reaches 10%.^7^ Many liver cancer patients are unable to be cured due to tumor metastasis. High recurrence and metastasis are the main reasons that affect the prognosis and cause the death of HCC patients.^8^ Clinically, serum detection of alpha‐fetoprotein (AFP) and B‐ultrasound are commonly used to detect HCC and HCC postoperative metastasis and recurrence.^9^ Unfortunately, both methods have limitations. Therefore, to improve the survival rate and prognosis of HCC patients, it is necessary to study the mechanism of invasion and metastasis of HCC cells, explore more effective biomarkers for early diagnosis and HCC metastasis prediction, and identify target molecules for intervention therapy.

The levels of LAIR‐1, a type I transmembrane glycoprotein widely expressed in most hematopoietic cells, differ markedly in the process of cell differentiation and activation and inversely regulate immune responses and cell differentiation.^10^ Recently, increasing evidence has shown that LAIR‐1 is also widely distributed in solid cancer cells. For example, LAIR‐1 overexpression has been detected in oral squamous cell carcinoma, cervical cancer, and ovarian cancer.^11^, ^12^, ^13^ The studies have indicated that the LAIR‐1 level was closely related to the pathological differentiation of tumors. These findings indicated that the LAIR‐1 might have relevant effects on nonhematopoietic tumor development and is a broad‐spectrum tumor suppressor. Therefore, the mechanism of tumor regulation by LAIR‐1 is worthy of further study.

Recently, we observed that LAIR‐1 expression was detected in HCC but not in healthy liver tissues, and high LAIR‐1 expression was related to unfavorable cancer differentiation.^14^ Furthermore, LAIR‐1 overexpression was closely related to the decreased overall survival of HCC patients.^14^ However, the role of LAIR‐1 in HCC progression and its molecular mechanism remains unclear. Thus, the mechanism of HCC tumor regulation by LAIR‐1 deserves further investigation.

The PI3K‐AKT‐mTOR is a critical cellular signaling pathway that regulates cell biological behaviors, such as cell proliferation, migration, and invasion. The PI3K‐AKT‐mTOR pathway is abnormally activated in various malignant tumors, including HCC, bladder, and endometrial cancer, and is considered the primary pathway for tumor cell survival.^15^, ^16^, ^17^ Some studies have found that nearly 50% of HCC cells have overexpression of the proteins relevant to the PI3K‐AKT‐mTOR pathway.^15^ The downregulation of these proteins can notably decrease HCC cell proliferation and migration and has been a focus of intense investigation for clinical application.^18^ Some research has shown that LAIR‐1 can decrease ovarian cancer cell proliferation and migration via PI3K‐AKT‐mTOR pathway suppression.^13^ However, there is no relevant literature report on the relationship between the PI3K‐AKT‐mTOR pathway and LAIR‐1 in HCC cells. Based on the research background, we hypothesized that LAIR‐1 might affect HCC cell proliferation and invasion via PI3K‐AKT‐mTOR pathway regulation.

## 2 MATERIALS AND METHODS

### 2.1 Cells and culture conditions

Healthy human hepatocyte L02 and HCC cells, including HepG2, Bel‐7402, MHCC97‐H, and Huh‐7 were purchased from The Cell Bank of Type Culture Collection (Chinese Academy of Sciences). The cell lines were cultivated in Dulbecco's Modified Eagle Medium (DMEM), supplemented with penicillin (100 U/mL), streptomycin (100 μg/mL), and fetal bovine serum (FBS) (10%) in a 5% CO_2_ atmosphere at 37°C.

### 2.2 Reagents and antibodies

Anti‐LAIR‐1 antibody [lc12] was obtained from Abcam. The primary antibodies, such as PI3K (9272S), AKT (9272S), p‐AKT (4060S), mTOR (2983S), p‐mTOR (5536S), and GAPDH (10494‐1‐AP) were all purchased from the Cell Signaling Technology. The secondary antibodies conjugated with horseradish peroxidase (HRP) were also purchased from the CST. The PI3K inhibitor (LY294002) was provided by MedChem Express.

### 2.3 RNA interference

The negative control and LAIR‐1 small interfering RNA (siRNA) were provided by Santa Cruz Biotechnology. For siRNA transfection, MHCC97‐H cells were cultivated in six‐well plates in a nonantibiotic medium the day before transfection. Once cells reached 60%–80% confluence, the transfection was performed through Lipofectamine 3000 and medium without serum. After 4 h, the transfection medium replaced with a culture medium supplemented with FBS (10%), and cells were incubated under 5% CO_2_ at 37°C.

### 2.4 Lentivirus infection

Commercially available lentiviral (LV)‐LAIR‐1 constructs (Tianyucheng Biotechnology) were modified to overexpress LAIR‐1. The lentivirus was transfected into Huh‐7 cells, and cells transfected with an empty vector served as the negative control.

### 2.5 MHCC97‐H and Huh‐7 cell grouping

MHCC97‐H cells were grouped in control (normal culture), siRNA group (LAIR‐1‐transfected negative control), LAIR‐1 siRNA (transfected with LAIR‐1 siRNA), and LAIR‐1 siRNA + PI3K inhibitor (transfected with LAIR‐1 siRNA and treated with the PI3K inhibitor LY294002).

Huh‐7 cells were divided into the control group (normal culture), the NC group (cells transfected with empty vector), and the OE group (LAIR‐1‐lentivirus).

### 2.6 Immunocytochemistry

When the cells reached 80% confluence on glass coverslips and were fixed with preheated 4% paraformaldehyde for 25 min at room temperature. The coverslips were immersed in citrate buffer (pH = 6.0) (95°C–98°C, 5 min) and then incubated in 3% H_2_O_2_ (room temperature, 15 min) for antigen retrieval. Cells were blocked with 1% BSA (serum albumin, Beyotime Biotechnology) at room temperature for 20 min and then incubated with monoclonal mouse anti‐human LAIR‐1 (ab14826; Abcam) overnight at 4°C. The cells were washed with PBS and incubated for 30 min with biotinylated anti‐mouse secondary antibody (BA1001; Boster Biological Technology, Ltd.) at 37°C. Streptomycin anti‐biotin‐peroxidase solution was added to each coverslip and incubated for 30 min at room temperature. Diaminobenzidine (DAB) was used for color development. Cells were counterstained with Mayer's hematoxylin.

### 2.7 Western blot (WB)

Cell lysates were obtained using radioimmunoprecipitation assay (RIPA) lysis buffer. The bicinchoninic acid (BCA) protein assay kit (Beyotime) was used to quantify protein. The proteins (40–80 μg) were separated using sodium dodecyl sulfate‐polyacrylamide gel electrophoresis (SDS‐PAGE), transferred onto a polyvinylidene difluoride (PVDF) membrane, and blocked with skim milk (5%). The membranes were incubated with primary antibodies overnight at 4°C and then with HRP‐coupled secondary antibodies for 2 h at room temperature. Protein bands were revealed using an enhanced chemiluminescence (ECL) kit, following the manufacturer's instructions. The GAPDH protein was used as the loading control. The images were obtained through Molecular Imager and analyzed using Quantity One‐4.6.5.

### 2.8 Quantitative real‐time polymerase chain reaction (qRT‐PCR)

Relative levels of expression of LAIR‐1 were detected using qRT‐PCR. Total RNA was extracted using TRIzol reagent (Invitrogen). cDNA was obtained by using reverse transcription RNA and amplified by PCR. The expression levels of GAPDH mRNA were measured as the internal control. The sequences of primers were as follows: LAIR‐1: forward, 5′‐TTCTGTCCTTGCATTGGTGC‐3′ and reverse, 5′‐GAAAGTCACATGGCTCCCCA‐3′; GAPDH: forward, 5′‐ATGGGCAGCCGTTAGGAAAG‐3′ and reverse, 5′‐ATCACCCGGAGGAGAAATCG‐3′. Reactions were carried out in triplicate. The relative LAIR‐1 mRNA expression levels were calculated by the 2^−ΔΔCT^ algorithm.

### 2.9 Cell viability

Cells were inoculated into 96‐well plates (1 × 10^4^ cells/well) after transfection. CCK‐8 was used for determining hepatoma cell viability. The absorbance at 450 nm was measured and the data obtained were displayed as a percentage of the control.

### 2.10 Cell invasion assay

Transfected MHCC97‐H or Huh‐7 cells were harvested, and cell invasion assay was performed using Transwell chambers covered with Matrigel, following the manufacturer's protocol (BD Biosciences Cat. No. 354480). Cells were re‐suspended in DMEM without serum (2 × 10^5^ cells/mL), and the lower chamber was added a culture medium with FBS. After 48 h, the cells that remained in the upper chamber were cleared using a cotton swab. The cells in the lower chamber were fixed using paraformaldehyde and stained with crystal violet. The invasive cells were observed and counted under an inverted microscope.

### 2.11 Colony formation assay (CFA)

For CFA, cells were inoculated into 6‐well plates (500 cells/well) and incubated in a damp 5% CO_2_ atmosphere at 37°C for two weeks. Next, the colonies were fixed with paraformaldehyde, stained with crystal violet, and then counted.

### 2.12 Data analysis

All data analyses were conducted using GraphPad Prism 7. The Log‐rank (Mantel‐Cox) test was applied to complete Kaplan–Meier Survival analysis. The results were statistically assessed through one‐way or two‐way analysis of variance along with Bonferroni post hoc tests. Three independent experiments were conducted, and the data obtained were displayed as mean ± standard error of the mean (SEM). Differences were statistically significant when *p* < .05.

## 3 RESULTS

### 3.1 Detection of LAIR‐1 level in HCC cells

**Figure 1:**
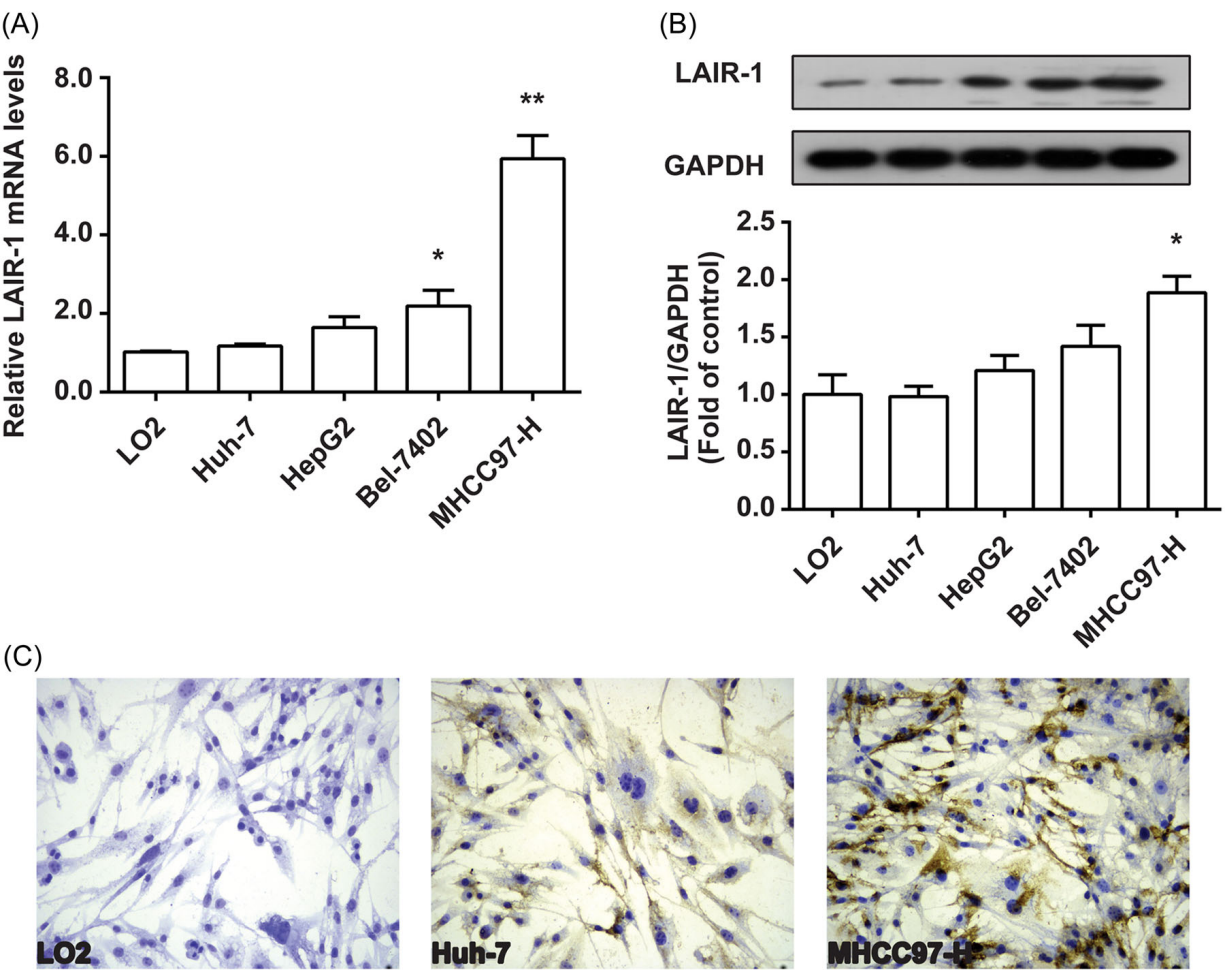
Detection of LAIR‐1 level in hepatoma cells. (A) qRT‐PCR analysis showed the different expression level of LAIR‐1 mRNA in four HCC cell lines and normal hepatocytes. (B) WB showed different LAIR‐1 protein expressions in HCC cells and normal hepatocytes. (C) Immunocytochemistry was utilized to detect the expression and location of LAIR‐1 in cells LO2, Huh‐7, and MHCC97‐H. Magnification 400×. Results demonstrated that MHCC97‐H cells had the highest LAIR‐1 expression. Three independent experiments were conducted and the results obtained were displayed as mean ± SEM (*p < .05, **p < .01 vs. LO2 group). HCC, hepatocellular carcinoma; qRT‐PCR, quantitative real‐time polymerase chain reaction; WB, western blot.

Expression of LAIR‐1 in healthy human hepatocyte LO2 and four HCC cell lines (HepG2, Bel‐7402, MHCC97‐H, and Huh‐7) were analyzed by qRT‐PCR and western blot analysis. qRT‐PCR results showed that Bel‐7402 and MHCC97‐H cells exhibited a higher LAIR‐1 mRNA expression level compared with three other cell lines (Figure 1A). The LAIR‐1 protein expression levels in the HCC cells HepG2, Bel‐7402, MHCC97‐H, and Huh‐7 were significantly higher than that of healthy hepatocyte LO2 cells (Figure 1B). Immunocytochemical staining assay showed that LAIR‐1 was not expressed in healthy human hepatocyte LO2, weakly expressed in Huh‐7, and strongly positively expressed in MHCC97‐H (Figure 1C). LAIR‐1 mainly expressed on the cell cytoplasm.

### 3.2 LAIR‐1 knockdown facilitated the viability, colony formation, and invasion of MHCC97‐H cells

**Figure 2:**
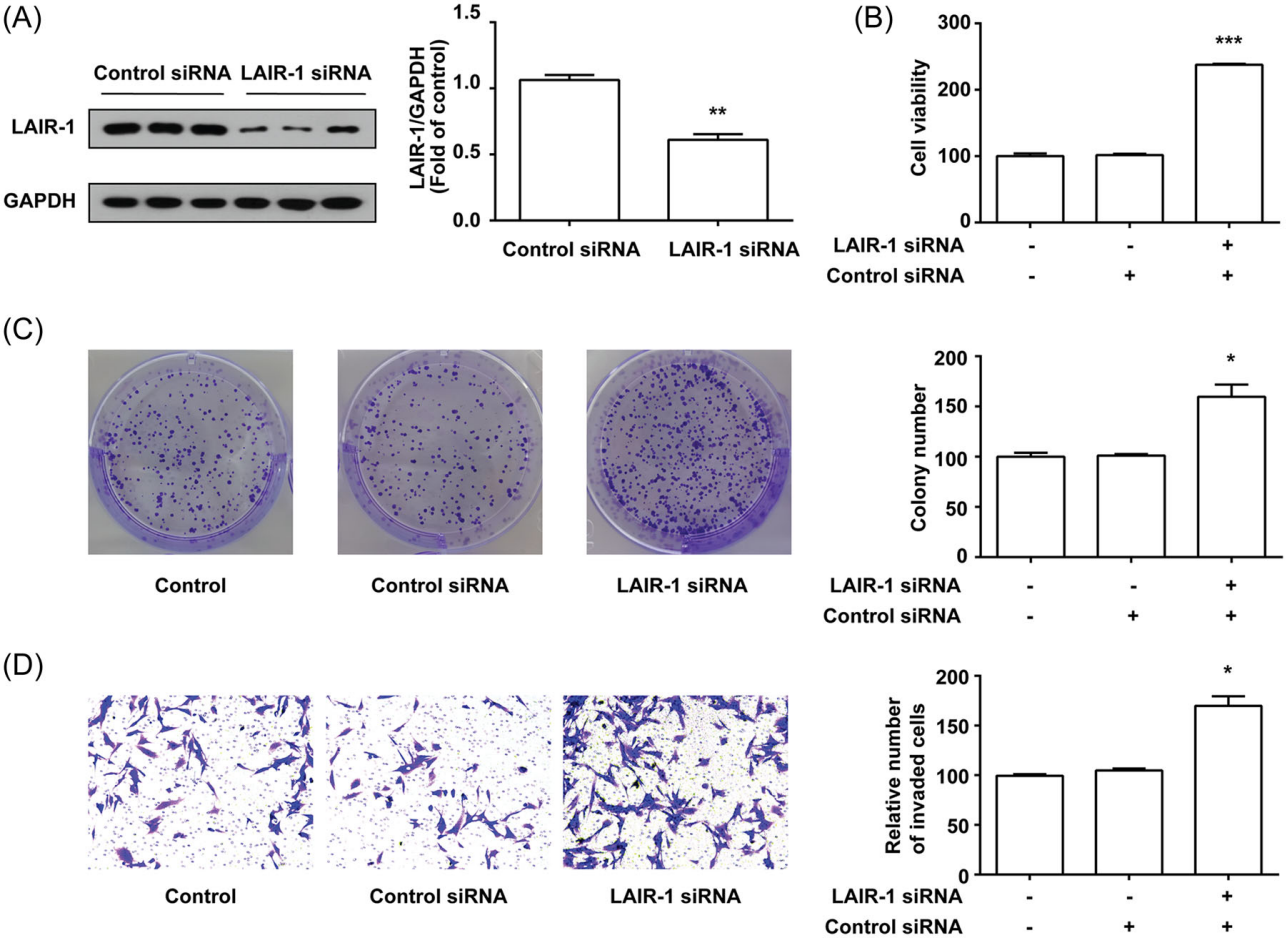
LAIR‐1 knockdown increased MHCC97‐H cell viability, colony formation, and invasion. (A) The LAIR‐1 knockdown efficiency was assessed through WB. (B) CCK‐8 assay was performed to measure cell viability. (C) CFA was used to determine the clonogenic ability of MHCC97‐H cells. (D) Transwell assay was used to examine the influence of LAIR‐1 knockdown on cell invasion. Three independent experiments were conducted, and the data obtained were displayed as mean ± SEM. Magnification 100×. No statistical difference in the migratory and invasive abilities was detected between the control and control siRNA groups (*p* > .05). (*p < .05, **p < .01, ***p < .001 vs. control and control siRNA groups). CCK‐8, Cell Counting Kit‐8; CFA, colony formation assay; SEM, standard error of mean; WB, western blot.

Among all HCC cell lines, MHCC97‐H cells exhibited the highest LAIR‐1 protein expression level (Figure 1B). Thus, MHCC97‐H cells were selected for subsequent experiments. LAIR‐1 siRNA was used to further validate the influence of LAIR‐1 on MHCC97‐H cells in vitro. WB was utilized to confirm the LAIR‐1 knockdown efficiency (Figure 2A).

Cells are major tumor components and exhibit powerful proliferation capability. Tumor cells can easily migrate to other parts of the and release damaging substances, harming healthy organ structures and threatening human health and life.^19^ Thus, exploring the potential mechanism of tumor cell proliferation and invasion is critical in suppressing cancer occurrence and progression.^20^ The results of the CCK‐8 assay showed that MHCC97‐H cell viability increased significantly (Figure 2B). CFA was also conducted to investigate the anti‐proliferation effect of LAIR‐1. The LAIR‐1 gene expression suppression in MHCC97‐H cells by LAIR‐1 siRNA dramatically improved the clonogenic ability of MHCC97‐H cells (Figure 2C). Cell invasion assay demonstrated that MHCC97‐H cells knocked down for LAIR‐1 were more invasive than the cells in the control and siRNA control groups (Figure 2D). Therefore, endogenous LAIR‐1 might have a critical role in HCC development.

### 3.3 LAIR‐1 inhibited Huh‐7 cells viability, colony formation, and invasion

**Figure 3:**
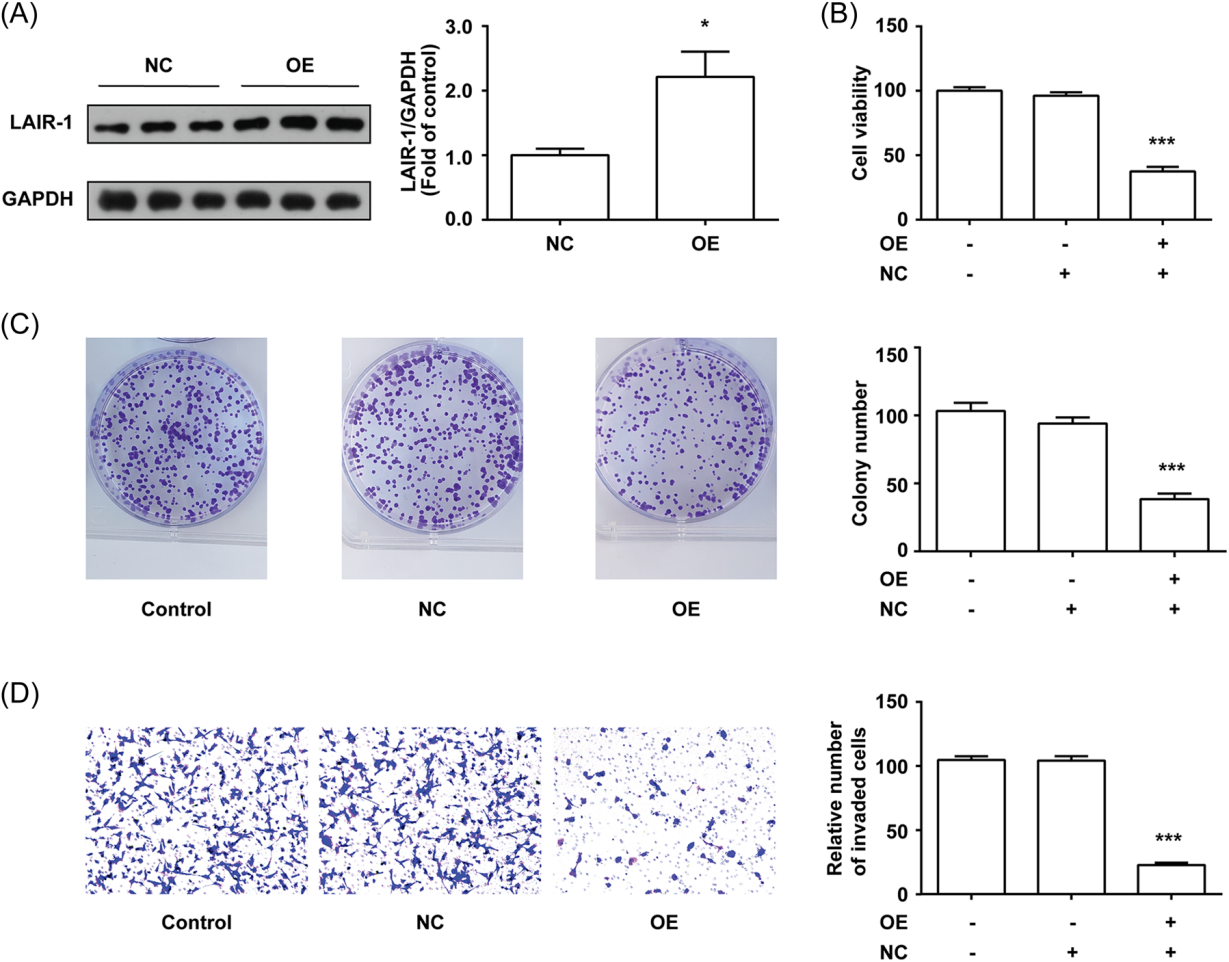
LAIR‐1 inhibited Huh‐7 cells viability, colony formation, and invasion. (A) Western blot was used to confirm the efficiency of LAIR‐1 overexpression in Huh‐7 cells. GAPDH was used as a loading control. (B and C) The cell growth was measured by CCK‐8 and CFA. (D) Cell invasion ability was assessed by transwell assay. Three independent experiments were conducted, and the data obtained were displayed as mean ± SEM. Magnification 100×. No statistical difference in the migratory and invasive abilities was detected between the control and NC groups (*p* > .05). (*p < .05, ***p < .001 vs. control and NC group). CCK‐8, Cell Counting Kit‐8; CFA, colony formation assay; SEM, standard error of mean.

In Figure 2, WE showed that the knockdown of LAIR‐1 promoted MHCC97‐H cells viability, colony formation, and invasive ability. We confirmed the effect of LAIR‐1 on the malignant phenotypes of other HCC cells by further stably overexpressing LAIR‐1 in Huh‐7, a HCC cell line with lower level LAIR‐1 expression as shown in Figure 1. The overexpression of LAIR‐1 was confirmed by WB (Figure 3A). Cell viability assay using a CCK‐8 kit revealed that overexpression of LAIR‐1 significantly inhibited Huh‐7 cells viability (Figure 3B). We also performed a CFA to verify the antiproliferative activity of LAIR‐1. As shown in Figure 3C, LAIR‐1 overexpression significantly reduced the clonogenic ability of Huh‐7 cells. Next, we examined whether LAIR‐1 could affect the invasion properties of Huh‐7 cells in vitro. The transwell assay showed that LAIR‐1 suppressed Huh‐7 cell invasion (Figure 3D). These data, combined with Figure 2, indicated that LAIR‐1 has an anti‐tumorigenic role in HCC cells.

### 3.4 LAIR‐1 suppressed HCC cells by regulating the PI3K‐AKT‐mTOR axis

HCC has a high metastasis and recurrence rate and strong invasiveness.^21^ The PI3K‐Akt‐mTOR pathway overactivation was detected in HCC.^15^ The PI3K‐Akt‐mTOR pathway contains three main proteins, namely PI3K, Akt, and mTOR, and their overactivation can further promote HCC proliferation and metastasis.^18^ Some research has reported that LAIR‐1 could regulate the proliferation and apoptosis of various malignancies through the PI3K‐Akt‐mTOR pathway.^11^, ^12^, ^13^

**Figure 4:**
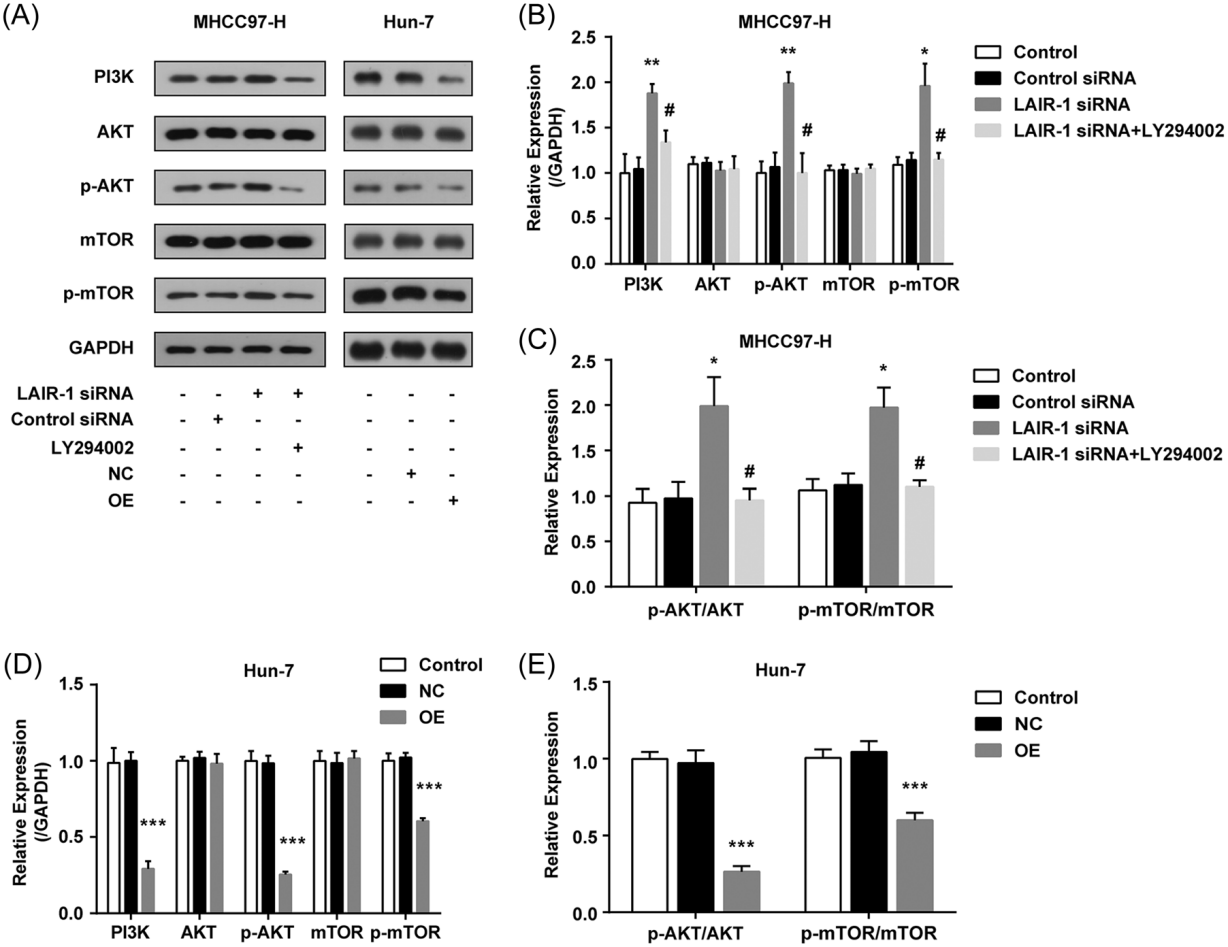
LAIR‐1 suppressed HCC cells by regulating the PI3K‐AKT‐mTOR axis. (A) WB analyses of PI3K, AKT, p‐AKT, mTOR, and p‐mTOR levels in each group. (B–D) The protein quantification was performed based on the band gray values. Three independent experiments were conducted, and the data obtained were displayed as mean ± SEM. No statistical difference between the control and control siRNA group or NC group (*p* > .05) was detected. (*p < .05, **p < .01, ***p < .001 vs. Control siRNA grou*p* or NC group; ^#^ *p* < .05 vs. LAIR‐1 siRNA + PI3K inhibitor group). HCC, hepatocellular carcinoma; NC, negative control; siRNA, small interfering RNA; SEM, standard error of mean.

In comparison to the control group, no obvious distinction was detected in the control siRNA group or NC group (*p* > .05). WB indicated that the levels of PI3K, p‐AKT and p‐mTOR in MHCC97‐H cells were upregulated after LAIR‐1 siRNA knockdown (Figure 4A,B), whereas they were significantly decreased after LAIR‐1 overexpressing in Huh‐7 cells (Figure 4A,D). LAIR‐1 knockdown in MHCC97‐H cells dramatically improved the ratio of p‐AKT/AKT and p‐mTOR/mTOR (Figure 4C). In contrast, the ratio of p‐AKT/AKT and p‐mTOR/mTOR decreased significantly in LAIR‐1 overexpressing Huh‐7 cells (Figure 4E).

We further investigated whether PI3K‐AKT inhibitor could offset the influence of LAIR‐1 on MHCC97‐H cells treated with LY294002. The combination of LAIR‐1 knockdown and LY294002 treatment showed stronger PI3K inhibition and AKT and mTOR phosphorylation levels compared to LAIR‐1 knockdown alone (Figure 4A–C), consistent with the effect of overexpressing LAIR‐1 in Huh‐7 cells (Figure 4A,D,E). These findings suggest that LAIR‐1 can modulate the PI3K‐AKT‐mTOR pathway activity in HCC cells.

## 4 DISCUSSION

LAIR‐1 belongs to the immunosuppressive receptor family, mostly expressed in hematopoietic cells, and plays a negative role in regulating the function of hematopoietic cells.^10^ Previous studies have shown that LAIR‐1 might be associated with hematopoietic tumor occurrence and development.^14^ Nonetheless, reports on LAIR‐1 expression in nonhematopoietic cells and tumor cells are rare, and its mechanism of action has not been fully clarified. Recently, it was found that the LAIR‐1 protein and mRNA expression levels in HCC tissue were dramatically higher than that in adjacent tissue, and LAIR‐1 was remarkably correlated with the pathological grade, T stage, and age of HCC patients.^14^ These results indicated that LAIR‐1 affects HCC development. Nevertheless, the molecular mechanism of LAIR‐1 in regulating HCC occurrence and development has not been reported.

This study indicated that LAIR‐1 expression was detected in normal hepatocytes and hepatoma cells. The LAIR‐1 protein expression in MHCC97‐H cells with high metastatic potential was notably higher than that in low metastatic potential cells (LO2, HepG2, Bel‐7402, and Huh‐7). The metastatic potential of the cells decreases, and the LAIR‐1 expression directly correlate (Figure 1), suggesting that the LAIR‐1 level is closely related to HCC invasion and metastasis.

Tumor recurrence still occurs in 70% of HCC cases in 5 years because of extrahepatic or intrahepatic metastasis caused by a multicentric occurrence or primary lesion,^22^ even after successful surgical resection and antiviral drug treatment. The recurrence and metastasis after surgeries are prevalent and can cause main obstacles to prolonged patient survival. Metastasis is associated with multiple processes in carcinogenesis, such as cancer cell colony dissemination, invasion, survival, and proliferation.^23^ The malignant proliferation of hepatoma cells, strong invasion, and migration make up the foundation and premise of tumor diffusion and metastasis and are also the major triggers for further tumor disease progression.^24^ Therefore, metastasis and recurrence are the primary reasons for unfavorable HCC prognosis.^25^ Thus, investigating the relevant mechanism underlying hepatoma cell proliferation and invasion may positively affect clinical HCC suppression and mitigation. For transfection experiments, we screened separately the LAIR‐1 highest expression cell line MHCC97‐H and the lowerest expression cell line Huh‐7 (Figure 1A–C) TO establish stable knockdown (MHCC97‐H) and overexpression (Huh‐7) cell lines. The results suggested that after LAIR‐1 expression silencing in MHCC97‐H cells, the MHCC97‐H cell viability, colony formation, and invasion were significantly enhanced (Figure 2B–D), whereas they were significantly decreased after LAIR‐1 overexpressing in Huh‐7 cells (Figure 3B‐D). These data were consistent with the previous studies that LAIR‐1 could dramatically suppress HCC occurrence, development, and metastasis. Therefore, further study of the molecular mechanism of LAIR‐1 in HCC might provide a novel target for HCC diagnosis and treatment.

The fundamental feature distinguishing malignant tumors from benign ones is their susceptibility to metastasis.^26^ Many factors can suppress HCC by inactivating the PI3K‐AKT‐mTOR pathway,^27^, ^28^, ^29^ indicating that the PI3K‐AKT‐mTOR pathway plays a pivotal role in HCC occurrence, progression, and migration. mTOR, AKT, and PI3K inhibitors are still considered attractive and potential therapeutic alternatives for HCC treatment.^30^ Nonetheless, the relevant molecular mechanisms of these inhibitors on HCC have not been elucidated. In both MHCC97‐H and Huh‐7 cells, WB analyses of PI3K, p‐AKT, AKT, p‐mTOR, and mTOR indicated an association between the expression of LAIR‐1 and the regulation of the PI3K‐AKT‐mTOR axis. To further demonstrate that LAIR‐1 was related to PI3K‐AKT‐mTOR pathway regulation in HCC, we treated the LAIR‐1 siRNA group with the PI3K inhibitor LY294002. LY294002 effectively offset the influence of silencing LAIR‐1 expression on the LAIR‐1 siRNA group and reduced expressions of p‐AKT, p‐ mTOR, and PI3K (Figure 4A,B). Moreover, the ratios of p‐AKT/AKT and p‐mTOR/mTOR were also decreased (Figure 4C).

We previously mentioned that LAIR‐1 overexpression in HCC tissues was significantly associated with worse overall survival.^14^ The Wu et al. study^14^ does not contradict our present results showing that overexpression of LAIR‐1 suppresses HCC cell viability, colony formation, and invasion and indicates that LAIR‐1 may play a negative regulatory role in HCC. In the tumor microenvironment, regarding LAIR‐1 cross‐linking with its collagen or other ligands, it leads to a decrease in immune activity, along with a decline in T‐cell function,^31^, ^32^ thereby worsening the overall survival.

In summary, this study first indicated that LAIR‐1 overexpression was detected in HCC cells, and the downregulation of LAIR‐1 levels increased HCC cell viability, colony formation, and invasion, which might be associated with the PI3K‐AKT‐mTOR signaling pathway suppression. The Wu et al. study^14^ and our findings suggested that LAIR‐1 affects HCC development. These findings might help to understand better the mechanism of LAIR‐1 affecting the biological function of HCC cells. We provide a new alternative for clinical diagnosis and treatment of HCC recurrence and metastasis. Thus, LAIR‐1 might become a novel target for HCC diagnosis and treatment.

## AUTHOR CONTRIBUTIONS

**Ti Zhou**: Conceptualization; data curation; formal analysis; project administration; writing—original draft; writing—review & editing. **Luqing Liu**: Data curation; formal analysis; software. **Haibin Lan**: Data curation; formal analysis; software. **Donglin Fang**: Conceptualization; data curation; methodology; writing—original draft; writing—review & editing.

## CONFLICT OF INTEREST STATEMENT

The authors declare that there is no conflict of interest.

## ETHICS STATEMENT

This article does not contain any studies with human participants or animals performed by any of the authors.

## Data Availability

All data generated or used during the study appear in the submitted article.
